# Acaricidal and Repellent Effects of Essential Oils against Ticks: A Review

**DOI:** 10.3390/pathogens10111379

**Published:** 2021-10-26

**Authors:** Sidi Mohammed Ammar Selles, Mokhtaria Kouidri, Marta G. González, Julia González, María Sánchez, Azucena González-Coloma, Jaime Sanchis, Latifa Elhachimi, A. Sonia Olmeda, José Maria Tercero, Félix Valcárcel

**Affiliations:** 1 Institute of Veterinary Sciences, University of Tiaret, Tiaret 14000, Algeria; mokhtariakouidri@yahoo.fr; 2 Laboratory of Research on Local Animal Products, University of Tiaret, Tiaret 14000, Algeria; 3 Laboratory of Farm Animal Products, University of Tiaret, Tiaret 14000, Algeria; 4 Grupo de Parasitología Animal, Departamento de Reproducción Animal (INIA-CSIC), 28040 Madrid, Spain; marta.glez.degano@gmail.com (M.G.G.); maria2985@hotmail.com (M.S.); sanchisjaime@gmail.com (J.S.); valcarcel.felix@inia.es (F.V.); 5 Center for Vector Biology, Department of Entomology, Rutgers University, New Brunswick, NJ 08901, USA; julia.gonzalez@rutgers.edu; 6 Villamagna S.A., Finca “La Garganta”, 14440 Villanueva de Córdoba, Spain; jmtercero@fincalagarganta.com; 7 Instituto de Ciencias Agrarias (ICA), Consejo Superior de Investigaciones Científicas (CSIC), 28006 Madrid, Spain; azu@ica.csic.es; 8 Facultad de Veterinaria, CENUR Litoral Norte, Universidad de la República, Rivera, Salto 1350, Uruguay; 9 Département de parasitologie et de Santé Publique, Institut Agronomique et Vétérinaire Hassan II, Rabat B.P. 6202, Morocco; latifa.elhachimi@gmail.com; 10 Departamento de Sanidad Animal, Facultad de Veterinaria, Universidad Complutense de Madrid, 28040 Madrid, Spain; angeles@ucm.es

**Keywords:** ticks, natural compounds, efficacy, standardization

## Abstract

Tick control is a priority in order to prevent the transmission of vector-borne diseases. Industrial chemical acaricides and repellents have been the most efficient tools against hard ticks for a long time. However, the appearance of resistances has meant the declining effectiveness of the chemicals available on the market. The trend today is to develop alternative control methods using natural products to replace nonefficient pesticides and to preserve the efficient ones, hoping to delay resistance development. Traditional in vitro evaluation of acaricidal activity or resistance to synthetic pesticides have been reviewed and they mainly focus on just one species, the one host tick (*Rhipicephalus (Boophilus) microplus* (Acari: Ixodidae)). Recent reports have called for the standardization of natural product components, extraction techniques, and experimental design to fully discover their acaricidal potential. This study reviews the main variables used in the bibliography about the efficiency of natural products against ticks, and it proposes a unification of variables relating to ticks, practical development of bioassays, and estimation of ixodicidal activity.

## 1. Introduction

Ticks are forced bloodsucker ectoparasites belonging to the Order Ixodida, which comprises three families: Ixodidae (hard ticks, 720 species), Argasidae (soft ticks, 186 species), and Nuttalliellidae (1 species) [1,2]. They are also one of the main groups of disease vectors, and tick-borne diseases (TBDs) have long been recognized as one of the major constraints to livestock development in various countries [3], particularly in the cattle industry in tropical and subtropical regions [4]. Likewise, they are the most devastating as they cause huge economic losses [5]. These losses are estimated in the billions of US dollars [4], as a consequence of higher production costs due to constant antiparasitic treatments [6] and the effects generated by the infestation: blood loss, reduced weight gain and milk production, and skin damage at the site of attachment [7].

Many commercially available chemicals are used in current tick control strategies: arsenicals, chlorinated hydrocarbons, carbamates, macrocyclic lactones [8,9], organophosphates, formamidines, pyrethroids [8,9,10,11], fluazuron, and fipronil [10,11]. They have generally been sprinkled on, poured on, or injected into animals, with high costs for farmers [10,11].

Unfortunately, the misuse, overuse, and inappropriate application of chemical acaricides led to the development and selection of resistance in the tick population [4,7,12,13,14,15]. However, apart from their high costs, these acaricides could also be potentially hazardous through contamination of ruminant milk and meat [13,16,17] and thus, may have an effect on human health [18,19], as well as contamination of the environment with residues harmful to humans and animals [15,16,18,20,21]. Due to this and the growing interest in organic farming practices, several acaricides have undergone restriction of use in the global market, such as organochlorines, organophosphates, and pyrethroids [18]. Consequently, the development of new agents and/or effective alternative strategies for their control is necessary [6,22]. Therefore, many other current strategies used for control of resistant tick populations such as biological control using pathogens or predators, pheromone-assisted control, herbal pour-on or dip preparations including green manufactured nanoparticles, and vaccination, as well as design of acaricide resistance mitigation programs based on integrated pest management control [3,23,24].

Among these alternative strategies, plant-derived products can be promising acaricidal product sources, especially essential oils [6,7,15,23,24]. The essential oil’s acaricidal activity is due to the variability of its chemical composition and the relationship between these compounds [25]. Moreover, given their low toxicities and their solubility in water [25] these compounds can contribute to the production of milk and animal meat free from dangerous chemicals which are harmful to humans, animals, and the environment [13]. This review aims to present an overview of tick control methods, tick resistance, essential oils in control of ticks, and their mechanisms of action.

## 2. Materials and Methods 

The data obtained from the following databases were used for the realization of this work: Web of Science (WOS), Science Direct, Scielo, and PubMed (consulted from May to July 2021). We selected articles focusing on the acaricidal effect of EOs against ticks using the following keywords: “Essential oil”, “Ticks”, “Acaricidal”, “Repellent”, “Mode of action”, and “Resistance”.

Therefore, we considered the research items complying with at least one of the following criteria: (1) Stage/specie, (2) bioassay, (3) reproductive efficiency/acaricidal effect, (4) toxicity, (5) repellent effect, and (6) country.

Acaricidal classes were arranged in a table according to the first year of use, containing mode of action, first report of resistance, and mechanisms of resistance.

The plant species were arranged in a table in alphabetical order, also containing the stage and tick specie, bioassay, major compounds concentration, effect, LC_50_, LC_90,_ and country.

## 3. Ticks and Synthetic Acaricides

Arsenic and its derivatives were intensively used at the end of the 19th and the beginning of the 20th centuries because they had a short residual effectiveness time, were cheap, stable, and water-soluble [26]. When arsenic use decreased, due to the high toxicity of its residues, its prohibition forced the appearance of the first organochlorines: dichlorodiphenyltrichloroethane (DDT), benzene hexachloride (BHC), lindane, dieldrin, or toxaphene, which were used extensively throughout the twentieth century [27]. Initially, organochlorides were highly effective against ticks showing high residual effectiveness an ease of use, but the majority accumulated residues in the environment and animal tissues [26]. In the 1960s, organophosphates and carbamate derivatives complemented or replaced organochlorides. These new acaricides offered the advantage of being biodegradable and rapidly metabolized, although they also quickly lost their effectiveness [28]. In the 1970s formamidines appeared, such as amitraz, in an attempt to avoid the fast reduction in effectiveness of the preceding products [29]. From the 1980s, the use of acaricides with low toxicity in mammals proliferated, such as pyrethrins and some biopesticides (macrocyclic lactones: avermectins and milbemycins) [30]. The pyrethrins gave rise to the pyrethroids, synthetic analogs obtained by successive isosteric modifications [31], more stable and with less residual effect. Pyrethroids are classified as first-generation (allethrin), second-generation (tetramethrin, resmethrin, bioresmethrin, biolalethrin, and fontarin), third-generation (fenvalerate and permethrin), and the current fourth-generation, which includes cypermethrin and decamethrin, among others [32]. Finally, in the late twentieth century, mite growth regulators derived from benzoyl-phenyl urea (fluazuron, for example) began to be used [28], along with other chemical acaricides, phenylpyrazoles, such as fipronil. Spinosad is a relatively recent insecticide-acaricide produced from the fermentation of metabolites of the actinomycete bacterium *Saccharopolyspora spinosa* and a mixture of two components A and D spinosyn [33]. In both laboratory and field tests, those products were found to be equally effective against ticks and could therefore be used as an alternative [34,35]. The latest addition to the market for ectoparasiticides for pets is isoxazolines [36,37,38,39].

## 4. Tick Control Methods

Several methods are applied to combat ticks and tick-borne diseases [40]. At first, tick control was based on using a mixture of lard and sulfur, a combination of lard and kerosene, cottonseed oil or fish oil from kerosene mixtures, cottonseed oil and sulfur, 10% kerosene emulsion, a mixture of cottonseed oil and crude petroleum oil, or Beaumont crude oil [8,41]. Thereafter, the treatment of cattle by baths based on mineral oil and “carbolics” by Australian researchers occurred, continuing as recently as 1895 [8,42]. Currently, the main method to control ticks is the use of chemical acaricides [27,41].

Many acaricides are available on the market such as arsenicals, organochlorines, organophosphates, carbamates, formamidines, pyrethroids, macrocyclic lactones, phenylpyrazoles, benzoylphenylurea, spinosad, and isoxazolines (Table 1) [9,10,11,14,40,41]. The use of these acaricides reduces the tick population, as well as the diseases transmitted by ticks. However, the effectiveness of an acaricide depends on the activity of a product, as well the quality and quantity of active material sprayed or injected [40]. Moreover, inappropriate and long-term application of these acaricides, frequency of treatment, underdosing, and persistent use of a chemical group for tick control enables improvement of tolerance/resistance to the chemical and leads to the evolution of resistance in many tick species [4,14,40,41,43,44,45].

Several studies recommended an integrated strategy for the control of ticks in cattle based on the house management, slow-burning of the wastes near the walls of the animal sheds, pasture alternation and/or rotation, pasture burning, clearance of vegetation, nutritional management, rotation of acaricides, using combinations of acaricides, immunization via vaccination, improving genetic resistance in cattle, improving resistance diagnostic tests, biological control, and ethnoveterinary practices against ticks [14,41,46,47,48].

**Table 1:** Chemical acaricidal class, mode of action, and their mechanisms of resistance.

Acaricidal Class	First Year of Use	Mode of Action	First Report of Resistance	Mechanisms of Resistance	References
Arsenic	1893		1937		[9,41,49]
Organochlorines	1946	GABA-gated chloride channel antagonists Bind at the picrotoxinin site in the γ-aminobutyric acid (GABA) chloride ionophore complex.	1952	Enhanced metabolism and reduced absorption of the chemical	[14,40,41]
Organophosphates	1955	Acetylcholinesterase Inhibits the action of acetylcholinesterase	1965	Target-site insensitivity	[14,40,41]
Carbamates	1955	Inhibits the action of acetylcholinesterase	1965		[14,40,41]
Formamidines (Amitraz)	1975	Octopamine receptor α-2 agonist Overstimulates the nervous system	1981	Mutations in the octopamine/tyramine receptor Target-site insensitivity in G protein-coupled receptors Amino acid substitution in the beta-2-adrenergic-like octopamine receptor	[4,8,14,40,50]
Pyrethroids	1977	GABA-gated chloride channel antagonists Prolongs opening of sodium channels in nerve, muscle, and other excitable cells.	1989	Mutations in the voltage gated sodium channel gene	[8,40,41,51]
Macrocyclic lactones	1981	Has a toxic effect on ticks by stimulating the release and binding of GABA at nerve endings, which eventually blocks the transmittance of electrical activity in nerves and muscle cells Glutamate–gated Cl-channel Blocks nerve signals by interfering with the glutamate gated chloride (GlCl)	2001	Insensitivity of the GABA or glutamate gated chloride ion channels	[8,40,41,52]
Phenylpyrazoles (Fipronil)	1993	Blocks the gamma-Aminobutyric acid gated chloride ion-channel (GABA-C)	2003	Mutations in the GABA-Cl gene	[41,53,54,55]
Benzoylphenyl urea (Fluazuron)	1990	Disturbs cuticle formation Blocks the incorporation of radiola-beled N-acetylglucosamine	2010		[41,56]
Spinosad (Tetracyclic-macrolide compounds)	2001	Nicotinic acetylcholine receptors (nAChRs) γ-amino-butyric acid (GABA) receptors Hyperexcitation and disruption of an insect’s nervous system			[41,57,58,59,60]
Isoxazolines	2014	Inhibits GABA-gated chloride ion channels			[36,61,62]

## 5. Tick Resistance

Chemical products used in traditional tick control methods are at the center of eradication and control efforts because they offer relatively rapid and effective control of tick populations. However, resistance is the result of irrational and long-term use of acaricidal drugs. In addition, they are expensive and can be harmful to the environment and dangerous for consumers if the recommended withdrawal times for foods of animal origin are not respected [63,64,65]. Resistance is broadly defined as a change in the sensitivity of the target species to a drug [66,67]. The concept of drug resistance can be defined as “the ability of a strain of parasite to survive and/or multiply despite the administration and absorption of a drug administered to doses equal to or greater than those usually recommended but within the tolerance of the subject” (World Health Organization, 1965) or “the ability of some parasites to tolerate toxic doses of a drug that would be lethal to most of their congeners” [68,69].

There are several definitions of acaricide resistance and different types of resistance were observed. In parasitology, four types of resistance were defined:

- Natural resistance or tolerance: “present in the external body-part and in all individuals of the species and does not develop as a result of acaricidal use”. This may be due to the impermeability of the cuticle or behavioral traits. It is not necessarily transmitted to their offspring [47].
- Acquired resistance: is “resistance that results from heritable decreases in sensitivity to drugs over time ” [14,40].
- Cross-Resistance: is defined as “the sharing of resistance among different acaricides with a similar mode of action” [14,40].
- Multiple resistances: is defined as “a resistance to more than one drug, even though they have different modes of action” [14,40].

The first report of tick resistance, against arsenic, was due to the indiscriminate use of this product for more than 30 years (1890–1910) to control *Rhipicephalus (Boophilus) microplus* (Wharton 1983), the main tick affecting domestic cattle over the world. There are many reports describing resistances to formamidine, amitraz, permethrin [70,71,72,73], and even to the more modern fipronil [53]. Unfortunately, there are instances of resistances to practically all the synthetic acaricides as reviewed by [14]. Although negative consequences of resistances have been partially relieved with the rotation products with different mechanisms of action, it continues to be a major problem in large territories of Africa and America [48,74,75,76,77]. This is the main threat given that most of these acaricidal groups are still applied. The development of safe and effective new acaricidal agents is therefore of great interest.

## 6. Natural Products

Plant products containing bioactive metabolites represent a promising alternative for the control of ticks that are susceptible and/or resistant to conventional acaricides. Studies of the effects of essential oils and plant extracts against different classes of ticks showed efficacies of 5–100% [78]. Following the line of the search for ecological alternatives for effective tick control, products derived from microorganisms or natural products were defined as biopesticides [79]. The products derived from plants are particularly attractive due to their low toxicity, scarce environmental permanence, and the complex chemistry that hinders the development of the resistances. The use of natural products for the control of ticks offers advantages but still has certain limitations. The first disadvantage is the variability of the composition of certain products, such as essential oils, and therefore their effectiveness, for which the identification and subsequent standardization of the fractions and possible effective synergies are required. Another disadvantage could be the characteristics of the product, such as photosensitivity or high volatility, which limit the residual activity depending on the form of presentation [17]. Among the advantages, its role as an alternative in the control of resistant ticks stands out, its environmental innocuousness, and the minimum impact on animal and human health that facilitates its registration and subsequent commercialization [80]. At the beginning of the 19th century, the Caucasian and Persian tribes used pyrethrum flowers as a method of control against body lice [31]. Pyrethrum or Dalmatian pyrethrum (*Tanacetum cinerariifolium* or *Chrysanthemum cinerariaefolium*) is an evergreen plant of the Asteraceae family, with insecticidal properties of low toxicity to mammals. Another plant derivative traditionally used is the neem tree (*Azadirachta indica*), belonging to the Meliaceae family that originates in the Indian subcontinent. For centuries, Indians have relied on this tree to strengthen their health and remedy dozens of diseases; in addition, it has been used to protect stored food and as a natural fertilizer and pesticide for the fields, since it intervenes in the feeding of arthropods and the hormonal processes of their development [81,82]. It is now valued throughout the world as an important source of phytochemicals for use in human health and pest control. Neem oil contains at least 100 biologically active compounds [83]. Essential oils are naturally produced by plants as secondary compounds, which are obtained for commercial use by various forms of distillation, and plant extracts are obtained through various forms of solvent extraction; some of them stand out for their pesticide, growth-regulating, and repellent or dissuasive properties [17].

## 7. Essential Oils in Control of Ticks

### Acaricidal Activity of Essential Oils against Ticks

In recent decades, natural products and their compounds have been the most productive source for new drug development. Among them, essential oils and isolated terpenoids have shown activity against diverse stages of several species of ticks [63]. Many studies have reported the ovicidal (inhibited oviposition and inhibited hatchability), effects against all the stages of ticks [3,14,34,84,85,86,87].

The variability in the chemical composition of essential oils and the relationship between compounds play an important role in acaricidal activity. It is difficult to attribute the observed biological effects to the major chemical compounds of essential oils. These biological effects can be attributed to a synergistic action between the minority compounds and other minor or major molecules [15]. Additionally, the substances obtained from plants have a low cost, few residual effects, and a low incidence of generating resistance [15,88].

Many researchers have studied the acaricidal effects of essential oils against ticks of the Ixodidae family. Table 2 summarizes the studies carried out on this subject.

## 8. Mechanisms of Action of the Essential Oils and/or Their Components against Ticks

Essential oils are the most studied plant-derived compounds for tick control and prevention [9,16,18,122]. Two effects of essential oils against ticks were observed: acaricidal or repellent effects [9,16,122,123,124,125,126,127]. They cause various effects against ticks: feeding inhibition [125,128,129], inhibition of chitin synthesis [9,78,129], decrease in growth, development, or reproduction [9,78,125,126,128,130], and affect tick behavior [129].

Several studies have reported that essential oils act against ticks through three modes of action: neurotoxicity effect [16,127,129,131,132], cytotoxicity effect [127,133], and mechanical effects [129,132,134].

### 8.1. Neurotoxicity Effects

Three mechanisms of the induced action-neurotoxic acaricide effect of essential oils have been described: inhibition of acetylcholinesterase (AChE) [124,127,129,132,135], antagonism with receptors for the neurotransmitter octopamine [124,127,136] and action on GABA [16,124,135,136]. However, there are controversial data on the insecticidal essential oils action on GABA. Some authors have attributed this effect to chloride channel closure by gamma-aminobutyric acid (GABA) [16,135] or to an increase in Cl current induced by the neurotransmitter GABA [124,136].

Salman et al. [127] reported that the neurotoxic effect of essential oils as acaricides acted on motor function. This is due to an increase produced on catecholamines in the central nervous system inhibiting the activity of monoamine oxidase.

### 8.2. Inhibition of Acetylcholinesterase Activity 

Acetylcholine is an important neurotransmitter in both the central nervous system (CNS) and the peripheral nervous system of many organisms including arthropods, whereas, acetylcholinesterase is an enzyme controlling the concentration of the excitatory neurotransmitter acetylcholine (ACh) in the synaptic cleft [124]. This is one of the most important enzymes in the neuroneuronal and neuromuscular junction [136]. 

Many works have shown that essential oils as well as several different terpenoid compounds exhibited an anti-AChE activity [124,135,136,137,138]. Several components were tested on AChE of arthropods: α-pinene and β-pinene, β-phellandrene, carvacrol, limonene, menthol, menthone, 1,8-cineole, cis-ocimene, niloticin, eucalyptol, pulegone, linalool, citral, bornyl acetate, p-cymene, γ-terpinene. It has been shown that these components are effective with doses varying from nM to μM concentrations [139,140,141,142,143,144,145]. 

Furthermore, Terpinen-4-ol, a monoterpenoid found at high concentrations in tea tree oil, inhibits arthropod acetylcholinesterase [129]. In addition, Camilo et al. [136] have shown that carvacrol has an AChE inhibitory effect 10-fold higher than its thymol isomer. This effect is linked to the position of the hydroxyl group in its structure that plays a key role. 

### 8.3. Binding-Octopamine Receptors

Octopamine is a multifunctional naturally occurring biogenic amine and plays a key role as a neurotransmitter, neurohormone and neuromodulator in invertebrate systems, with a physiological role analogous to that of noradrenaline in vertebrates [135,146]. In arthropods, the group of biogenic amine messengers consists of five members: dopamine, tyramine, octopamine, serotonin, and histamine [124]. Octopamine and tyramine modulate various functions such as the metabolism and behavior of arthropods [127]. The neurotoxic and cytotoxicity activities of essential oils and/or their purified constituents (eugenol, α- terpineol, and cinnamic alcohol) against arthropods are probably attributable to binding to tyramine and octopamine receptors resulting in lethal effects [124,127,147]. 

The essential oils act as agonists of octopamine receptors. They cause an increase in both the level of cAMP and the level of intracellular Ca^2+^. Likewise, they induce the activation of PKA and PKC kinases and the phosphorylation of many proteins (including ion channels, enzymes and receptors) [135,148].

### 8.4. Mechanical Effects

Several studies have cited the mechanical effects of essential oils on parasites. It is the hydrophobic nature of essential oils that is responsible for this effect. Death from water stress or suffocation is the result of disruption of cuticular waxes and blockage of respiratory stigmas [129,132,134].

Moreover, essential oils are known for their antihistamine [85,132] and anti-inflammatory effect. They reduce inflammation by increasing the production of interleukin-10 [132,149,150,151].

### 8.5. Repellent Effects 

Many plants and/or their essential oils have a repellent effect against arthropods [122,126,152,153].

The majority of synthetic molecules with repellent effects pose problems for their safety, efficacy, and environmental impacts [153,154,155,156,157,158]. Therefore, the use of friendly and biodegradable natural acaricides have attracted the attention of researchers [159]. Due of this, plants or their derivatives constitute an interesting alternative to fight against ticks [122,125,153,157,160]. 

Repellents are substances that act locally or remotely, which prevent the landing, stings, or bites of arthropods. Halos et al. [161] defined repellents as “chemicals which cause arthropods to perform oriented movements away from their source”. There are two types of repulsion, repulsion in the strict sense, where an irritant effect by direct contact can be observed. This effect causes the tick to drop off before attaching itself to the host. The second action is known as sensu lato repellency, which causes attachment inhibition or detachment of already attached ticks [129].

The mechanism of action of the repellent effect of essential oils is poorly understood [162]. These act by producing a vapor barrier that deters the arthropod from coming into contact with the skin or landing on the skin [123,127,162,163]. 

Several studies reported the repellent activity of essential oil. Essential oils of *Cupressus funebris*, *Juniperus communis*, and *Juniperus chinensis* have an *Amblyomma americanum* nympha repellency with an EC_50_ of 0.426, 0.508, and 0.917 mg oil/cm^2^ filter paper, respectively. However, the essential oils of *Cupressus funebris* had a repellent effect against *Ixodes scapularis* with an EC_50_ of 0.103 mg oil/cm^2^ filter paper [164]. Wanzala et al. [152] showed that *Tagetes minuta* essential oil had more repellent activity than that of *Tithonia diversifolia* essential oil against *Rhipicephalus appendiculatus* with a repellent dose at 0.5 probabilities of 0.0021 mg and 0.263 mg, respectively. Likewise, 5% oregano and spearmint essential oils exhibited as natural clothing repellents against *Ixodes ricinus* comparable to 20% DEET for 24 h [153].

Jaenson et al. [165] found that the *Corymbia citriodora* essential oil showed a 100% repellent effect against *Ixodes ricinus* nymphs. Moreover, lavender and geranium essential oil, when diluted to 30% in 1,2-propanediol, had 100% of repellency activity.

Moreover, Kulma et al. [166] noted a moderate to high repellency activity at 65 to 85% against *Ixodes ricinus* females 5 min after the application of *Lavender* and *Eucalyptus* essential oils.

The volatile nature of essential oils is a major drawback because their activities usually dissipate relatively quickly [123,127,162,167]. For this reason, several fixatives are used to improve the duration of repellency of essential oils such as mustard and coconut oils, genapol, ethanol, polyethylene glycol, liquid paraffin petroleum jelly, and salyciluric acid [127].

## 9. Conclusions

The past decade was characterized by a growing interest from researchers to study the effectiveness of essential oils against ticks.

Numerous preclinical studies have documented the acaricidal efficacy of EOs and/or their main compounds, in many cases elucidating their mechanism of action and lethal dose, and their relative biosafety and biodegradability in nature, which may constitute a serious alternative to the use of chemical acaricides.

Unfortunately, there are hardly any clinical studies and more in vivo research is needed to standardize the experimental design, to establish the correct doses to administer in animals, and to determine the synergistic and antagonistic effects, as well as to study the toxicological profile of these EOS in mammals.

Finally, given the volatile nature of many essential oils, it is necessary to further develop their formulation as acaricides (microencapsulation and nanoformulation) to maximize the contact time of these essential oils with ticks, and to avoid problems related to residual activity and the possible phytotoxicity of certain compounds used at high concentrations.

## Figures and Tables

**Table 2:** Essential oils (EOs) tested against ticks. Complete information about major compounds, concentrations, effect, LC50, LC90, and country is provided in the Supplementary File (Table S1).

Origin	Stage/Specie	Bioassay	Ref.
*Acmella oleracea*	L/N *A. sculptum*	LPT; LIT; NPT; NIT	[89]
*Allium sativum*	L/*R. microplus*	LPT	[24]
*Aloe rupestris*	L/A *R. decoloratus*	LPT; ACT; AIT	[90,91]
*Alpinia zerumbet*	L/EF *R. microplus*	LPT; AIT	[15]
*Antizoma angustifolia*	L/*R. decoloratus*	LPT	[90]
*Arisaema anurans*	L/EF/egg *R. microplus*	LIT; AIT; EHT	[92]
*Artemisia annua*	EF/*R. microplus*	In vivo	[93]
*Artemisia dracunculus*	L/*H. lusitanicum*	LCT	[94]
*Artemisia herba alba*	Egg/L/N/*H. aegyptium*; L/*H. lusitanicum*	EPT; LPT; NPT; LCT	[94,95]
*Calpurnia aurea*	L/*R. decoloratus*; A/*R. turanicus*	LPT; ACT; AIT	[90,91,96]
*Cananga odorata*	N/*I. ricinus*	OFPM; LETM	[97]
*Cedrus atlantica*	EF/*R. microplus*	AIT	[6]
*Chenopodium ambrosioides*	Egg/L/N *H. aegyptium*	EPT; LPT; NPT	[95]
*Cinnamomum verum*	L/EF *R. microplus*	LPT; AIT	[98]
*Cissus quadrangularis*	L/*R. decoloratus*; A/*R. turanicus*	LPT; ACT; AIT	[90,91]
*Citrus hystrix*	L/*R. microplus*	LIT	[24]
*Clematis brachiata*	L/*R. decoloratus*; A/*R. turanicus*	LPT; ACT; AIT	[90,91]
*Cleome gynandra*	L/*R. decoloratus*; A/*R. turanicus*	LPT; ACT; AIT	[90,91]
*Cuminum cyminum*	L/*R. microplus*	LPT	[23]
*Cymbopogon citratus*	EF/L *R. microplus*	AIT; LIT	[6,24]
*Cymbopogon martinii*	EF/*R. microplus*	AIT	[6]
*Dorystoechas hastata*	L/*R. turanicus*	LIT	[85]
*Eucalyptus camaldulensis*	L/A *H. scupense*	LIT; AIT	[3]
*Eucalyptus globulus*	L/A/*H. scupense*	LIT; AIT	[3]
*Ficus sycomorus*	L/*R. decoloratus*; A/*R. turanicus*	LPT; ACT; AIT	[90,91]
*Geranium macrorrhizum*	L/*H. lusitanicum*	LCT	[99]
*Homemade ocimum gratissimum*	L/*R. microplus*	LPT	[100]
*Hyssopus officinalis*	L/*H. lusitanicum*	LCT	[94,101]
*Illicium verum*	N/*I. ricinus*; A/*D. nitens*	OFPM; LETM; AIT	[97,102]
*Juniperus thurifera var. africana*	Egg/L/N *H. aegyptium*	EPT; LPT; NPT	[95]
*Laurus nobilis*	L/EF *R. microplus*	LPT; AIT	[103]
*Lavandula angustifolia*	L/*H. lusitanicum*	LCT	[94]
*Lavandula intermedia super*	L/*H. lusitanicum*	LCT	[101]
*Lavandula luisieri*	L/*H. lusitanicum*	LCT	[104]
*Lavandula pedunculata subsp. atlantica*	Egg/L/N *H. aegyptium*	EPT; LPT; NPT	[95]
*Lavandula stoechas*	L/A *H. scupense*	LIT; AIT	[3]
*Lippia gracilis*	L/EF/*R. microplus*	LIT; AIT; LST	[13]
*Lippia graveolens*	L/*R. microplus*	LPT	[24]
*Lippia sidoides*	L/EF *D. nitens*; L/EF *R.* *microplus*; L/N *R. sanguineus*; L/N *A. cajennense*	AIT; NPT	[86,105]
*Mangifera indica*	L/*R. microplus*; L/*H.* *anatolicum*; L/*Ha. bispinosa*	LPT	[106]
*Mentha longifolia*	L/*R. turanicus*	LIT	[85]
*Mentha piperita*	L/*H. lusitanicum*	LCT	[94]
*Mentha spicata*	L/*H. lusitanicum*	LCT	[94]
*Mentha suaveolens*	L/*H. lusitanicum*	LCT	[94]
*Mentha suaveolens subsp. timija*	Egg/L/N *H. aegyptium*	EPT; LPT; NPT	[95]
*Mesosphaerum suaveolens*	L/EF *R. microplus*	LPT; AIT	[15]
*Monsonia angustifolia*	L/*R. decoloratus*; A/*R. turanicus*	LPT; ACT; AIT	[90,91]
*Nemuaron vieillardii*	L/*R. microplus*	LPT	[107]
*Neoglaziovia variegata*	EF/*R. microplus*	AIT	[108]
*Ocimum gratissimum*	L/EF *R. microplus*; L/*A. sculptum*; L/*R. sanguineus*	LPT; AIT; LIT	[15,100,109]
*Ocimum urticaefolium*	L/*R. microplus*	LPT	[100]
*Ocotea elegans*	L/EF *R. microplus*	LPT; AIT	[110]
*Origanum floribundum*	L/A *H. scupense*	LIT; AIT	[3]
*Origanum minutiflorum*	A/*R. turanicus*	VPT	[34]
*Origanum onites*	A/*R. turanicus*	ACT	[111]
*Origanum vulgare subsp. virens*	L/*H. lusitanicum*	LCT	[94]
*Pelargonium luridum*	L/A *R. decoloratus*	LPT; ACT; AIT	[90,91]
*Pimenta dioica*	L/*R. microplus*	LPT	[23]
*Piper amalago*	L/*R. microplus*	LIT	[112]
*Piper corcovadensis*	L/*R. microplus*	LPT	[113]
*Piper mikanianum*	L/*R. microplus*	LIT	[112]
*Piper xylosteoides*	L/*R. microplus*	LIT	[112]
*Rosmarinus officinalis*	L/*R. microplus*; L/A *H. scupense*; L/*H. lusitanicum*	LPT; LIT; AIT; LCT	[3,24,94]
*Santolina chamaecyparissus*	L/*H. lusitanicum*	LCT	[100]
*Satureja calamintha*	Egg/L/N *H. aegyptium*	EPT; LPT; NPT	[95]
*Satureja montana*	L/*H. lusitanicum*	LCT	[94]
*Satureja thymbra*	A/*H. marginatum*	VPT	[84]
*Schinus molle*	L/EF *R. sanguineus*	LPT; AIT	[114]
*Schkuhria pinnata*	L/A *R. decoloratus*; A/*R. turanicus*	LPT; ACT; AIT	[90,91]
*Sclerocarya birrea*	L/*R. decoloratus*; A/*R. turanicus*	LPT; ACT; AIT	[90,91]
*Senecio adenotrichius*	L/*H. lusitanicum*	LCT	[115]
*Senna italica*	L/*R. decoloratus*; A/*R. turanicus*	LPT; ACT; AIT	[90,91]
*Syzygium aromaticum*	L/A *R. microplus*	LPT; AIT	[87]
*Tabernaemontana elegans*	L/*R. decoloratus*; A/*R. turanicus*	LPT; ACT; AIT	[90,91]
*Tagetes minuta*	EF/*R. microplus*	In vivo	[116]
*Tanacetum vulgare*	L/*H. lusitanicum*	LCT	[94]
*Tetradenia riparia*	L/EF *R. microplus*	LPT; AIT	[117]
*Thymus capitatus*	L/A *H. scupense*	LIT; AIT	[3]
*Thymus mastichina*	L/*H. lusitanicum*	LCT	[94]
*Thymus sipyleus subsp. sipyleus*	L/*R. turanicus*	LIT	[85]
*Thymus vulgaris*	L/*H. lusitanicum*	LCT	[94]
*Thymus zygis*	L/*H. lusitanicum*	LCT	[94]
*Zanthoxylum caribaeum*	A/*R.* *microplus*	AIT	[11]
plant not specified	L/*A. sculptum*; L/*D. nitens*; L/A *R. microplus*	LPT;LIT; AIT	[118,119,120]
Essentria IC3 ©	N/A *A. americanum*	NPT; APT	[121]
Mosquito Barrier ©	N/A *A. americanum*	NPT; APT	[121]
Vet’s Best ©	N/A *A. americanum*	NPT; APT	[121]
Wondercide ©	N/A *A. americanum*	NPT; APT	[121]

Stage: L: Larvae; N: Nymph; A: Adult; EL: Engorged larvae; EN: Engorged nymph; EF: Engorged females; UEL: Unengorged larvae; UEN: Unengorged nymphs. Specie: A.: Amblyomma; D.: Dermacentor; H.: Hyalomma; I.: Ixodes; Ha.: Haemaphysalis; R.: Rhipicephalus. R.microplus also includes R.(B.) microplus and R. decoloratus includes R.(B.) decoloratus. Bioassay: ACT: Adult contact test; AIT: Adult immersion test; APT: Adult packet test; FIT: Female immersion test; LCT: Larval contact test; LETM: Limited exposure time method; LIT: Larval immersion test; LPT: Larval packet test; LST: Larval sensitivity test; NPT: Nymphal packet test; OFPM: Open filter paper method; VPT: Vapor phase toxicity. Effect: CM Corrected mortality; CR: Control of reproduction; EE: efficiency of the extract; EMR: egg mass reduction; EPI: Egg production index; H: Hatching; IH: Inhibited hatchability; IO: Inhibited ovoposition; IR: Inhibited reproduction; M: Mortality; REI: Reproduction efficiency index.

## Data Availability

Not applicable.

## Supplementary Materials

The following are available online at https://www.mdpi.com/article/10.3390/pathogens10111379/s1, Table S1: Acaricidal effects of essential oils (EOs) and/or their major compounds against ticks.
