# Supplementary material for: Acaricidal and Repellent Effects of Essential Oils against Ticks: A Review
Source: Pathogens. 2021 Oct 26;10(11):1379. doi: 10.3390/pathogens10111379 (PMC8617816; doi:10.3390/pathogens10111379)
Supplement: Supplementary file 1 [file pathogens-10-01379-s001.zip › pathogens-1396705-supplementary.pdf]

Table S1. Acaricidal effects of essential oils (EOs) and/or their major compounds against ticks. **Stage**: L: Larvae; N: Nymph; A: Adult; EL: Engorged larvae; EN: Engorged nymph; EF: Engorged females; UEL: Unengorged larvae; UEN: Unengorged nymphs. **Specie**: A.: *Amblyomma*; D.: *Dermacentor*; H.: *Hyalomma*; I.: *Ixodes*; Ha.: *Haemaphysalis*; R.: *Rhipicephalus*. *R.microplus* also includes *R. (B.) microplus* and *R. decoloratus* includes *R.(B.) decoloratu*. **Bioassay**: ACT:Adult contact test; AIT: Adult immersion test; APT: Adult packet test; FIT: female immersion test; LCT: larval contact test; LETM: Limited exposure time method; LIT: Larval immersion test; LPT: Larval packet test; LST: larval sensitivity test; NPT: nymphal packet test; OFPM: Open filter paper method; VPT: Vapor phase toxicity. **Effect**: CM Corrected mortality; CR: Control of reproduction; EE: efficiency of the extract; EMR: egg mass reduction; EPI: Egg production index; H: Hatching; IH: Inhibited hatchability; IO: Inhibited ovoposition; IR: Inhibited reproduction; M: Mortality; REI: Reproduction efficiency index.

Origin	Stage/Specie	Bioassay	Major Compounds	Concentration	Effect	LC_{50}	LC_{90}	Country	Ref.
Acmella oleracea	L/ *A. sculptum*	LPT; LIT	methanol extract	0.4-50 mg/ml	100 % M	UEL 3.2 mg/ml EL 6.6 mg/ml		Brazil	[89]
	N/ *A. sculptum*	NPT; NIT	methanol extract	12.5-200 mg/ml	100 % M	38.5 mg/ml		Brazil	[89]
Allium sativum	L/ *R. microplus*	LPT	diallyl trisulfide, diallyl disulfide; methyl allyl trisulfide	5 %	100 % M			Mexico	[24]
Aloe rupestris	L/ *R. decoloratus*	LPT	acetone/ethanol extracts	1 %	CM % 26.3/3.14			South Africa	[90]
	A/ *R. turanicus*	ACT; AIT	acetone/ethanol extracts	200/12.5-100 mg/ml	CM % 0/20			South Africa	[91]
Alpinia zerumbet	L/ *R. microplus*	LPT	terpinen-4-ol; p-cymene; 1,8-cineole	100 mg/ml	100 % M	19.7 mg/ml		Brazil	[15]
	EF/ *R. microplus*	AIT	terpinen-4-ol; p-cymene; 1,8-cineole	100 mg/ml	98.2 % IO; IH	20.7 mg/ml		Brazil	[15]
Antizoma angustifolia	L/ *R. decoloratus*	LPT	acetone/ethanol extracts	1 %	CM % 1.9/10.6			South Africa	[90]
Arisaema anurans	L/ *R. microplus*	LIT	EO/asarone/cubenol; guaiol; eugenol; linalool; α-bisabolol	0.05-1.60 % w/v	65.0/73.2/49.6 % EE	0.147/0.115/ 0.338 %		China	[92]
	EF/ *R. microplus*	AIT	EO/asarone/cubenol; guaiol; eugenol; linalool; α-bisabolol	0.05-1.60 % w/v	36.3/44.2/17.7 % IO			China	[92]
	Egg/ *R. microplus*	EHT	EO/asarone/cubenol; guaiol; eugenol; linalool; α-bisabolol	0.05-1.60 % w/v	40.8/51.0/35.1 IH %	0.174/0.180 %/0.381 %		China	[92]
Artemisia annua	EF/ *R. microplus*	In vivo	artemisin	0.96 %	No efficacy			Brazil	[93]
Artemisia dracunculus	L/ *H. lusitanicum*	LCT	EO	40 μg/mg	30.20 % M	>40	>40	Spain	[94]
Artemisia herba alba	Egg/ *H. aegyptium*	EPT	camphor; cis-thujone; camphene	0.5-2 μl/ml	IH	1.105 μl/ml	2.409 μl/ml	Morocco	[95]
	L/ *H. aegyptium*	LPT	camphor; cis-thujone; camphene	0.5-2 μl/ml	M	0.775 μl/ml	1.509 μl/ml	Morocco	[95]
	N/ *H. aegyptium*	NPT	camphor; cis-thujone; camphene	0.5-2 μl/ml	M	0.0079 ml/cm^3	0.0182 ml/cm^3	Morocco	[95]
	L/ *H. lusitanicum*	LCT	EO	40 μg/mg	100 % M	0–40	20–40	Spain	[94]
Calpurnia aurea	L/ *R. decoloratus*	LPT	acetone/ethanol extracts	1 %	CM % 0.8/82.9			South Africa	[90]
	A/ *R. turanicus*	ACT; AIT	acetone/ethanol extracts	200/12.5-100 mg/ml	CM % 92.2/81.1			South Africa	[91]
	A/ *R. turanicus*	AIT	apigenin-7-o-β-d-glycoside/ isorhoifolin	3-100 mg/ml	M	1.35/0.65 mg/ml	3.95/1.71 mg/ml	South Africa	[96]
Cananga odorata	N/ *I. ricinus*	OFPM	industrial eo; ylang-ylang oil	0.05-0.2 μl oil/cm^2	M	0.185 μl/cm2	LC_{95} 0.385 $μl/cm^2$	Sweden	[97]
	N/ *I. ricinus*	LETM	star anise oil	0.4 μl oil/cm^2	M	0.1 $μl/cm^2$	LC_{95} 0.39 $μl/cm^2$	Sweden	[97]
Cedrus atlantica	EF/ *R. microplus*	AIT	α-himachalene	1 %	IH			Brazil	[6]

Chenopodium ambrosioides	Egg/ *H. aegyptium*	EPT	δ-3-carene; p-cymene; 1.4-epoxy-p-menth-2-ène	0.5-2 µl/ml	IH	1.672 µl/ml	2.409 µl/ml	Morocco	[95]
	L/ *H. aegyptium*	LPT	δ-3-carene; p-cymene; 1.4-epoxy-p-menth-2-ène	0.5-1 µl/ml	M	0.444 µl/ml	0.918 µl/ml	Morocco	[95]
	N/ *H. aegyptium*	NPT	δ-3-carene; p-cymene; 1.4-epoxy-p-menth-2-ène	0.5-2 µl/ml	M	0.0076 ml/cm^2	0.2367 ml/cm^2	Morocco	[95]
Cinnamomum verum	L/ *R. microplus*	LPT	EO/benzyl benzoate	0.5-25 mg/ml	M	1/3.37	LC_{95} 0.99-1.02/3.14-3.60	Brazil	[98]
	EF/ *R. microplus*	AIT	EO/benzyl benzoate	1.0-25 mg/mL	EPI; IO; REI; EE	60.78/45.7	LC_{95} 54.57-67.70/39.83-52.49	Brazil	[98]
Cissus quadrangularis	L/ *R. decoloratus*	LPT	acetone/ethanol extracts	1 %	CM % 17.2/80.4			South Africa	[90]
	A/ *R. turanicus*	ACT; AIT	acetone/ethanol extracts	200/12.5-100 mg/ml	CM % 6.67/11.1			South Africa	[91]
Citrus hystrix	L/ *R. microplus*	LIT	*α*- myrcene, linalool,; *α*-pinene, citronellal, neral, citronellol	30 %	100 % M	11.98 % (V/V)	24.84 % (V/V)	Malaysia	[24]
Clematis brachiata	L/ *R. decoloratus*	LPT	acetone/ethanol extracts	1 %	CM % 17.2/80.4			South Africa	[90]
	A/ *R. turanicus*	ACT; AIT	acetone/ethanol extracts	200/12.5-100 mg/ml	CM % 55.6/47.8			South Africa	[91]
Cleome gynandra	L/ *R. decoloratus*	LPT	acetone/ethanol extracts	1 %	CM % 15.3/28.9			South Africa	[90]
	A/ *R. turanicus*	ACT; AIT	acetone/ethanol extracts	200/12.5-100 mg/ml	CM % 66.7/77.8			South Africa	[91]
Cuminum cyminum	L/ *R. microplus*	LPT	cuminaldehyde, γ-terpinene; 2-caren-10-al	1.25 %	100 % M			Mexico	[23]
Cymbopogon citratus	EF/ *R. microplus*	AIT	geranial	1 %	IH			Brazil	[6]
	L/ *R. microplus*	LIT	linalool, eucalyptol, neral, camphene,; *α*-pinene, citronellal	30 %	100 % M	1.21 % (V/V)	6.28 % (V/V)	Malaysia	[24]
Cymbopogon martinii	EF/ *R. microplus*	AIT	geraniol	5 %	IH			Brazil	[6]
Dorystoechas hastata	L/ *R. turanicus*	LIT	EO	0.1 %	100 % M			Turkey	[85]
Eucalyptus camaldulensis	L/ *H. scupense*	LIT	p-cymene; spathulenol	6.25 µl/ml		0.207 µl/ml	1.653 µl/ml	Algeria	[3]
	A/ *H. scupense*	AIT	p-cymene; spathulenol	12.5 µl/ml	IO; IH; IR	0.207 µl/ml	1.653 µl/ml	Algeria	[3]
Eucalyptus globulus	L/ *H. scupense*	LIT	1,8-cineole; *α*-pinene	6.25 µl/ml		0.155 µl/ml	2.387 µl/ml	Algeria	[3]
	A/ *H. scupense*	AIT	1,8-cineole; *α*-pinene	12.5 µl/ml	IO; IH; IR	0.155 µl/ml	2.387 µl/ml	Algeria	[3]
Ficus sycomorus	L/ *R. decoloratus*	LPT	acetone/ethanol extracts	1 %	CM % 5.8/17.8			South Africa	[90]
	A/ *R. turanicus*	ACT; AIT	acetone/ethanol extracts	200/12.5-100 mg/ml	CM % 86.7/14.4			South Africa	[91]
Geranium macrorrhizum	L/ *H. lusitanicum*	LCT	β-elemenone, germacrone, linalool and thymol	20 mg/ml	100 % M	6.97	16.32	Spain	[99]
Homemade ocimum gratissimum	L/ *R. microplus*	LPT	(z)-β-ocimene, eugenol, β-caryophyllene, terpinen-4-ol	5 %	65.17 % M			New Caledonia	[100]
Hyssopus officinalis	L/ *H. lusitanicum*	LCT	industrial EO	40 µg/mg	0 % M			Spain	[101]
	L/ *H. lusitanicum*	LCT	EO	40 µg/mg	0 % M	>40	>40	Spain	[94]
Illicium verum	N/ *I. ricinus*	OFPM	industrial EO; ylang-ylang oil	0.05-0.2 µl/cm^2	M	0.185 µl/cm2	0.385 µl/cm^2	Sweden	[97]
	N/ *I. ricinus*	LETM	star anise oil	0.4 µl/cm^2	M	0.1 µl/cm^2	0.39 µl/cm^2	Sweden	[97]
	A/ *D. nitens*	AIT	1,8-cineole; camphor	40 mg/ml	11.2 % IH			Brazil	[102]
Juniperus thurifera var. africana	Egg/ *H. aegyptium*	EPT	sabinene	0.5-2 µl/ml	IH	1.449 µl/ml	2.692 µl/ml	Morocco	[95]
	L/ *H. aegyptium*	LPT	sabinene	0.5-1 µl/ml	M	0.513 µl/ml	1.551 µl/ml	Morocco	[95]
	N/ *H. aegyptium*	NPT	sabinene	0.5-1 µl/ml	M	0.0045 ml/cm^3	0.0118 ml/cm^3	Morocco	[95]

Laurus nobilis	L/ *R. microplus*	LPT	EO and eight fractions	4-200 μL/mL	M	0.13 μL/ml	LC_{99} 0.51 μL/ml	Brazil	[103]
	EF/ *R. microplus*	AIT	EO and eight fractions	4-200 μL/mL	EMR; IH			Brazil	[103]
Lavandula angustifolia	L/ *H. lusitanicum*	LCT	EO	40 μg/mg	100 % M	16.06	19.71	Spain	[94]
Lavandula intermedia super	L/ *H. lusitanicum*	LCT	industrial EO	40 μg/mg	90.93% M	28 μg/mg	38 μg/mg	Spain	[101]
Lavandula luisieri	L/ *H. lusitanicum*	LCT	camphor/5-methylen-2,3,4,4-tetramethylcyclopenten-2-one	40 μg/mg	100 % M	>162.5/53.8 μg/mg	-/84.4 μg/mg	Spain	[104]
Lavandula pedunculata subsp. atlantica	Egg/ *H. aegyptium*	EPT	camphor; α-pinene; camphene	0.5-2 μl/ml	IH	1.175 μl/ml	8.048 μl/ml	Morocco	[95]
	L/ *H. aegyptium*	LPT	camphor; α-pinene; camphene	0.5-1 μl/ml	M	0.505 μl/ml	1.014 μl/ml	Morocco	[95]
	N/ *H. aegyptium*	NPT	camphor; α-pinene; camphene	0.5-1 μl/ml	M	0.0036 ml/cm^3	0.0110 ml/cm^3	Morocco	[95]
Lavandula stoechas	L/ *H. scupense*	LIT	α- thujone; l-camphor	3.125 μl/ml	M	0.253 μl/ml	2.212 μl/ml	Algeria	[3]
	A/ *H. scupense*	AIT	α- thujone; l-camphor	6.25 μl/ml	IO; IH; IR	0.253 μl/ml	2.212 μl/ml	Algeria	[3]
Lippia gracilis	L/ *R. microplus*	LIT; AIT; LST	carvacrol	5 mg/ml	~100 % M	1.31- 4.34 mg/ml	2.18- 6.02 mg/ml	Brazil	[13]
	L/ *R. microplus*	LIT; AIT; LST	thymol	5 mg/ml	~100 % M	2.23 mg/ml	3.21 mg/ml	Brazil	[13]
	EF/ *R. microplus*	LIT; AIT; LST	carvacrol	5 mg/ml	~100 % M	6.55- 9.21 mg/ml	8.87- 20.01 mg/ml	Brazil	[13]
	EF/ *R. microplus*	LIT; AIT; LST	thymol	5 mg/ml	~100 % M	4.66 mg/ml	11.32 mg/ml	Brazil	[13]
	L/ *R. microplus*	LIT; AIT	EO/carvacrol/thymol	0.06-25 mg/ml	M	1.31/0.22 / 3.86 mg/ml	2.18/0.89/6.89 mg/ml	Brazil	[13]
	EF/ *R. microplus*	LIT; AIT	EO/carvacrol/thymol	1-25 mg/ml	M	4.66/4.46/5.50 mg/ml	11.32/5.71/6.01 mg/ml	Brazil	[13]
Lippia graveolens	L/ *R. microplus*	LPT	thymol, carvacrol, p-cymene; γ-terpinene	2.5 %	100 % M			Mexico	[24]
Lippia sidoides	L/ *D. nitens*	LPT	thymol; 1-monoestearin; 2-monopalmitin; carvacrol	2.5-20 μl/ml	100 % M	5.59 μl/ml		Brazil	[105]
	EF/ *D. nitens*	AIT	thymol; 1-monoestearin; 2-monopalmitin; carvacrol	10-80 μl/ml	0 % EPI			Brazil	[105]
	L/ *R. microplus*	LPT	thymol; 1-monoestearin; 2-monopalmitin; carvacrol	2.5-20 μl/ml	100 % M	11.13 μl/ml		Brazil	[105]
	EF/ *R. microplus*	AIT	thymol; 1-monoestearin; 2-monopalmitin; carvacrol	10-80 μl/ml	0 % EPI			Brazil	[105]
	L/ *R. sanguineus*	LPT	thymol; o-cymene; *e*-caryophyllene myrcene	14.10 mg/ml	99.5 % M			Brazil	[86]
	N/ *R. sanguineus*	NPT	thymol; o-cymene; *e*-caryophyllene myrcene	14.10 mg/ml	96 % M			Brazil	[86]
	L/ *A. cajennense*	LPT	thymol; o-cymene; *e*-caryophyllene myrcene	18.80 mg/ml	100 % M			Brazil	[86]
	N/ *A. cajennense*	NPT	thymol; o-cymene; *e*-caryophyllene myrcene	18.80 mg/ml	94 % M			Brazil	[86]
Mangifera indica	L/ *R. microplus*	LPT	aqueous extract/ tio(oh)2/synthesized tio2 nps	20-100 mg/l	M	94.22/70.99/ 13.21 mg/l		India	[106]
	L/ *H. anatolicum*	LPT	aqueous extract/ tio(oh)2/synthesized tio2 nps	20-100 mg/l	M	77.98/72.34/ 12.11 mg/l		India	[106]
	L/ *Ha. bispinosa*	LPT	aqueous extract/ tio(oh)2/synthesized tio2 nps	5-25 mg/l	M	80.54/69.40/ 10.64 mg/l		India	[106]
Mentha longifolia	L/ *R. turanicus*	LIT	EO	0.1 %	100 % M			Turkey	[85]
Mentha piperita	L/ *H. lusitanicum*	LCT	EO	40 μg/mg	100 % M	22.96	30.34	Spain	[94]
Mentha spicata	L/ *H. lusitanicum*	LCT	EO	40 μg/mg	100 % M	23.58	33.86	Spain	[94]
Mentha suaveolens	L/ *H. lusitanicum*	LCT	EO	40 μg/mg	100 % M	4.54	6.12	Spain	[94]

Mentha suaveolens subsp. timija	Egg/ *H. aegyptium*	EPT	menthone; pulegone	0.5-2 µl/ml	IH	0.910 µl/ml	1.465 µl/ml	Morocco	[95]
	L/ *H. aegyptium*	LPT	menthone; pulegone	0.5-2 µl/ml	M	0.990 µl/ml	1.889 µl/ml	Morocco	[95]
	N/ *H. aegyptium*	NPT	menthone; pulegone	0.5-2 µl/ml	M	0.408 ml/cm^3	0.1180 ml/cm^3	Morocco	[95]
Mesosphaerum suaveolens	L/ *R. microplus*	LPT	1,8-cineole; sabinene	100 mg/ml	87.7 % M	51.6 mg/ml		Brazil	[15]
	EF/ *R. microplus*	AIT	1,8-cineole; sabinene	100 mg/ml	IO/IH	31.3 mg/ml		Brazil	[15]
Monsonia angustifolia	L/ *R. decoloratus*	LPT	acetone/ethanol extracts	1 %	CM % 1.8/3.9			South Africa	[90]
	A/ *R. turanicus*	ACT; AIT	acetone/ethanol extracts	200/12.5-100 mg/ml	CM % 5.6/97.8			South Africa	[91]
Nemuaron vieillardii	L/ *R. microplus*	LPT	safrole; linalool; δ-cadinene; caryophyllene oxide; α-copaene	50 %	100 % M	14.67 %		New Caledonia	[107]
Neoglaziovia variegata	EF/ *R. microplus*	AIT	hexane extract	5-25 mg/ml	94.1 % IO 0.33 % H 99.81 % CR			Brazil	[108]
Ocimum gratissimum	L/ *R. microplus*	LPT	(commercial EO) eugenol, (z)-β-ocimene; germacrene d	5 %	85.20 % M			New Caledonia	[100]
	L/ *R. microplus*	LPT	γ-terpinene, thymol, p-cymene, α-terpinene, α-phellandrene, α-thujene	5 %	100 % M	0.98 %		Cameroon	[100]
	L/ *R. microplus*	LPT	eugenol; 1,8-cineole	100 mg/ml	99.4 % M	11.9 mg/ml		Brazil	[15]
	EF/ *R. microplus*	AIT	eugenol; 1,8-cineole	100 mg/ml	95.5 % IO 0.8 % IH	28.4 mg/ml		Brazil	[15]
	L/ *A. sculptum*	LIT	eugenol; eucalyptol (1,8-cineole)	20 mg/ml	100 % M	5.533 mg/ml		Brazil	[109]
	L/ *R. sanguineus*	LIT	eugenol; eucalyptol (1,8-cineole)	40 mg/ml	100 % M	6.253 mg/ml		Brazil	[109]
	L/ *R. microplus*	LIT	eugenol; eucalyptol (1,8-cineole)	20 mg/ml	100 % M	2.042 mg/ml		Brazil	[109]
Ocimum urticaefolium	L/ *R. microplus*	LPT	eugenol, β-bisabolene, elemicin	5 %	100 % M	0.90 %		Cameroon	[100]
Ocotea elegans	L/ *R. microplus*	LPT	EO	0.78-100 mg/ml	M	59.68 and 25.59 mg/ml		Brazil	[110]
	EF/ *R. microplus*	AIT	EO	6.25-100 mg/ml	IO; IH; REI	4.96 mg/ml	17.37 mg/ml	Brazil	[110]
Origanum floribundum	L/ *H. scupense*	LIT	carvacrol; p-cymene; γ-terpinene	3.125 µl/ml	IO; 0.98 % IH; IR	0.131 µl/ml	0.982 µl/ml	Algeria	[3]
	A/ *H. scupense*	AIT	carvacrol; p-cymene; γ-terpinene	6.25 µl/ml	IO; IH; IR	0.131 µl/ml	0.982 µl/ml	Algeria	[3]
Origanum minutiflorum	A/ *R. turanicus*	VPT	carvacrol	10 µl/ml	100 % M			Turkey	[34]
Origanum onites	A/ *R. turanicus*	ACT	carvacrol/linalool	6.25 and 12.5/25 %	100 % M	2.34 %/-	-/7.12 %	Turkey	[111]
Origanum vulgare subsp.virens	L/ *H. lusitanicum*	LCT	EO	40 µg/mg	100 % M	6.38	8.96	Spain	[94]
Pelargonium luridum	L/ *R. decoloratus*	LPT	acetone/ethanol extracts	1 %	CM % 9.5/10.5			South Africa	[90]
	A/ *R. turanicus*	ACT; AIT	acetone/ethanol extracts	200/12.5-100 mg/ml	CM % 38.9/38.9			South Africa	[91]
Pimenta dioica	L/ *R. microplus*	LPT	methyl eugenol, eugenol; 1,8-cineole	2.5 %	100 % M			Mexico	[23]
Piper amalago	L/ *R. microplus*	LIT	EO		M	2.33 µl/ml	LC_{99} 4.40 µl/ml	Brazil	[112]
Piper corcovadensis	L/ *R. microplus*	LPT	crude extract	1-40 mg/ml	96.63 % EE	>10 µg/mL	LC_{99} >10 µg/ml	Brazil	[113]
Piper mikanianum	L/ *R. microplus*	LIT	EO		M	2.33 µl/ml	LC_{99} 4.40 µl/ml	Brazil	[112]
Piper xylosteoides	L/ *R. microplus*	LIT	EO		M	6.15 µl/ml	LC_{99} 11.04 µl/ml	Brazil	[112]

Rosmarinus officinalis	L/ *R. microplus*	LPT	α- pinene, verbenone, 8-cineol	20 %	100 % M			Mexico	[24]
	L/ *H. scupense*	LIT	1,8-cineole; l-camphor; α-pinene; borneol l	1.562 µl/ml	95.72 % IO; IH; IR	0.108 µl/ml	0.495 µl/ml	Algeria	[3]
	A/ *H. scupense*	AIT	1,8-cineole; l-camphor; α-pinene; borneol l	3.125 µl/ml	IO; IH; IR	0.108 µl/ml	0.495 µl/ml	Algeria	[3]
	L/ *H. lusitanicum*	LCT	EO	40 µg/mg	100 % M	~10	~12	Spain	[94]
Santolina chamaecyparissus	L/ *H. lusitanicum*	LCT	industrial EO	40 µg/mg	98.61 % M	6.6	11	Spain	[101]
Satureja calamintha	Egg/ *H. aegyptium*	EPT	pulegone; menthone	0.5-2 µl/ml	IH	0.927 µl/ml	1.347 µl/ml	Morocco	[95]
	L/ *H. aegyptium*	LPT	pulegone; menthone	0.5-2 µl/ml	M	0.801 µl/ml	1.406 µl/ml	Morocco	[95]
	N/ *H. aegyptium*	NPT	pulegone; menthone	0.5-2 µl/ml	M	0.0072 ml/cm^3	0.02 ml/cm^3	Morocco	[95]
Satureja montana	L/ *H. lusitanicum*	LCT	EO	40 µg/mg	100 % M	4.68	8.33	Spain	[94]
Satureja thymbra	A/ *H. marginatum*	VPT	carvacrol; γ-terpinene	40 µl/ml	100 % M			Turkey	[84]
Schinus molle	L/ *R. sanguineus*	LPT	EO	0.125-2 %	M	0.21 %	0.80 %	Ecuador	[114]
	EF/ *R. sanguineus*	AIT	EO	0.125-20 %	29.6% IO 98.33% IH 77.40% IR			Ecuador	[114]
Schkuhria pinnata	L/ *R. decoloratus*	LPT	acetone/ethanol extracts	1 %	CM % 39.7/17.2			South Africa	[90]
	L/ *R. decoloratus*	LPT	acetone/ethanol extracts	1 %	CM % 0.0/12.6			South Africa	[90]
	A/ *R. turanicus*	ACT; AIT	acetone/ethanol extracts	200/12.5-100 mg/ml	CM % 88.9/86.7			South Africa	[91]
Sclerocarya birrea	L/ *R. decoloratus*	LPT	acetone/ethanol extracts	1 %	CM % 35.9/5.1			South Africa	[90]
	A/ *R. turanicus*	ACT; AIT	acetone/ethanol extracts	200/12.5-100 mg/ml	CM % 0/25.6			South Africa	[91]
Senecio adenotrichius	L/ *H. lusitanicum*	LCT	eremophilane sesquiterpenes 1/3/4/8	20 µg/mg	94/93/0/100 % M	5.2/8.24/-/2.46	19.56/14.12/-/10.96	Spain	[115]
Senna italica	L/ *R. decoloratus*	LPT	acetone/ethanol extracts	1 %	CM % 0.0/9.7			South Africa	[90]
	A/ *R. turanicus*	ACT; AIT	acetone/ethanol extracts	200/12.5-100 mg/ml	CM % 83.3/81.1			South Africa	[91]
Syzygium aromaticum	L/ *R. microplus*	LPT	EO/ eugenol	1-20 mg/ml	97.9/100 % M			Brazil	[87]
	A/ *R. microplus*	AIT	EO/ eugenol	12.5-100 mg/ml	100/100 % IH 100/100% EE			Brazil	[87]
Tabernaemontana elegans	L/ *R. decoloratus*	LPT	acetone/ethanol extracts	1 %	CM % 3.8/6.8			South Africa	[90]
	A/ *R. turanicus*	ACT; AIT	acetone/ethanol extracts	200/12.5-100 mg/ml	CM % 0/3.3			South Africa	[91]
Tagetes minuta	EF/ *R. microplus*	*In vivo*	EO (limonene, β-ocimene, dihydrotagetone and tagetone)	20 %	99.98 % EE			Brazil	[116]
Tanacetum vulgare	L/ *H. lusitanicum*	LCT	EO	40 µg/mg	23.37 % M	>40	>40	Spain	[94]
Tetradenia riparia	L/ *R. microplus*	LPT	14-hydroxy-9-epi-cariophyllene; α –cadinol limonene; ledol; fenchone; cis-muurolol-5-en-4-α-ol	25 % (w/v)	100 % M			Brazi	[117]
	EF/ *R. microplus*	AIT	14-hydroxy-9-epi-cariophyllene; α –cadinol limonene; ledol; fenchone; cis-muurolol-5-en-4-α-ol	1.8 % (w/v)	100 % M; IO; IH			Brazi	[117]
Thymus capitatus	L/ *H. scupense*	LIT	carvacrol	1.562 µl/ml	IO; IH; IR	0.058 µl/ml	0.358 µl/ml	Algeria	[3]

	A/ *H. scupense*	AIT	carvacrol	3.125 µl/ml	IO; IH; IR	0.058 µl/ml	0.358 µl/ml	Algeria	[3]
Thymus mastichina	L/ *H. lusitanicum*	LCT	EO	40 µg/mg	47.69 % M	>40	>40	Spain	[94]
Thymus sipyleus subsp. sipyleus	L/ *R. turanicus*	LIT	EO	0.1 %	100 % M			Turkey	[85]
Thymus vulgaris	L/ *H. lusitanicum*	LCT	EO	40 µg/mg	100 % M	5.52	9.52	Spain	[94]
Thymus zygis	L/ *H. lusitanicum*	LCT	EO	40 µg/mg	100 % M	2.44	3.88	Spain	[94]
Zanthoxylum caribaeum	A/ *R. microplus*	AIT	sylvestrene; muurola-4 (14),5-diene-trans; isodaucene; α-pinene	5 %	IO; IH			Brazil	[11]
	L/ *A. sculptum*	LPT	carvacrol/thymol/(e) -cinnamaldehyde	0.62-7.5 mg/ml		3.49/2.04/1.40		Brazil	[118]
	L/ *D. nitens*	LPT	carvacrol/thymol/(e) -cinnamaldehyde	0.31-7.5 mg/ml		2.33/2.17/1.68		Brazil	[118]
	L/ *R. microplus*	LPT;LIT	carvacrol/ethyl 2-methoxybenzoate/ethyl 2,5-dihydroxybenzoate	0.06-1 %	% M	59.72/0.91/30.32 µmol/ml		Mexico	[119]
	A/ *R. microplus*	AIT	carvacrol/ethyl 2-methoxybenzoate/ethyl 2,5-dihydroxybenzoate	50-250 ppm	27-2.34 % IO 15.59-6.03 % IH 27.97-2.98 % EE			Mexico	[119]
	L/ *R. microplus*	LPT	carvacrol/carvacrol acetate/thymol/thymol acetate/eugenol/eugenol acetate	0.312-15.0 mg/ml		0.83/2.49/1.26/2.97/2.77/4.25	2.02/4.21/2.21/8.52/5.35/13.10	Brazil	[120]
Essentria IC3 ©	N/ *A. americanum*	NPT	commertial product	0.19-100 %	~ 100 % M			USA	[121]
Essentria IC3 ©	A/ *A. americanum*	APT	commertial product	0.19-100 %	25–100 % M			USA	[121]
Mosquito Barrier ©	N/ *A. americanum*	NPT	commertial product	0.19-100 %	~ 100 % M			USA	[121]
Mosquito Barrier ©	A/ *A. americanum*	APT	commertial product	0.19-100 %	No effective			USA	[121]
Vet's Best ©	N/ *A. americanum*	NPT	commertial product	0.19-100 %	~ 100 % M			USA	[121]
Vet's Best ©	A/ *A. americanum*	APT	commertial product	0.19-100 %	No effective			USA	[121]
Wondercide ©	N/ *A. americanum*	NPT	commertial product	0.19-100 %	~ 100 % M			USA	[121]
Wondercide ©	A/ *A. americanum*	APT	commertial product	0.19-100 %	No effective			USA	[121]
